# “Peer support is the missing piece of the puzzle”: An evidence-based approach to developing peer support in Jigsaw-The National Centre for Youth Mental Health

**DOI:** 10.1192/j.eurpsy.2025.1621

**Published:** 2025-08-26

**Authors:** R. Murphy, A. Fitzgerald

**Affiliations:** 1 University College Dublin, Dublin, Ireland

## Abstract

**Introduction:**

In Ireland, 1 in 5 young adults (aged 18-25) experience moderate to severe levels of depression and anxiety. To meet the growing need for mental health supports, Jigsaw – The National Centre for Youth Mental Health – provides accessible, early intervention services throughout Ireland. Building on its reputation for championing youth voices and inspired by examples set by other international integrated youth mental health services, one such strategy Jigsaw is exploring is youth peer support.Peer support offers social, emotional, and practical assistance from young people with personal experience of mental health challenges. Evidence suggests that peer support can positively impact young people by promoting a recovery-based approach to mental health. However, despite the recognized benefits, peer support is underutilized in Irish youth mental health services, and there is limited guidance available for its development.

**Objectives:**

This research adopts a collaborative approach to intervention development, aiming to create an evidence-informed framework to guide the introduction of peer support in Jigsaw services.

**Methods:**

This PhD project adheres to the Medical Research Council’s Guidance for Developing and Evaluating Complex Interventions. The first stage of this project comprised a published scoping review of peer support in integrated youth mental health services and educational settings. The second stage of the project aimed to understand the benefits and challenges of peer support using semi-structured interviews with mental health professionals. The final stage of the project will take a participatory approach to intervention design, utilizing co-design workshops with stakeholders to identify potential intervention functions.

**Results:**

The scoping review identified common types of peer support programs (peer-delivered one-to-one support, self-help groups, and internet support groups) and target problems addressed (depression, anxiety, and psychological distress). Interviews with fifteen professionals revealed insights into the benefits of peer support for young people, such as increased connection and empowerment, and for services, such as reduced power imbalances and increased accessibility. Challenges for implementation were also identified, including boundary management, funding, and resource allocation. The ongoing final stage focuses on developing a program theory underpinning a potential peer support intervention in Jigsaw. Key stakeholders, including Jigsaw’s senior management and youth advisory panel (YAP), will be consulted to identify intervention components.

**Conclusions:**

Recommendations regarding key components of peer support, as well as barriers and facilitators to its implementation, will be shared to support other organizations in enhancing their understanding and application of peer support for youth mental health.

**Disclosure of Interest:**

None Declared

